# Low-Field MRI: A Paradigm Shift in Medical Imaging

**DOI:** 10.2174/0115734056397346250609162719

**Published:** 2025-06-20

**Authors:** Ayush Dogra

**Affiliations:** 1 Chitkara University Institute of Engineering and Technology, Chitkara University, Punjab, Rajpura, India

**Keywords:** Magnetic Resonance Imaging, Radiology, Diffusion-weighted imaging, Signal-to-noise ratio, Paediatric applications, acoustic noise

## 1 INTRODUCTION

Magnetic Resonance Imaging (MRI) has long been universally acknowledged as the gold standard for non-invasive diagnostic radiology, offering unparalleled soft tissue contrast and non-ionizing imaging capabilities. Nevertheless, its prevalent acceptance remains held up by exorbitant costs, infrastructure demands, and functional intricacies, especially in the absence of adequate resources [1]. The conventional high-field MRI machines, though offering superior resolution, also possess some significant constraints like inflated price tags, vulnerability to magnetic field distortions, and rigorous sitting conditions. The development of low-field MRI technology has emerged as a disruptive force, challenging conventional paradigms and unlocking new avenues and cost-effective imaging solutions [2]. Although these systems were previously unable to execute advanced techniques, such as Diffusion-Weighted Imaging (DWI), which limited their clinical utility, the recent rebirth of low-field MRI is driven by significant innovations, particularly the incorporation of Artificial Intelligence (AI) in image denoising and reconstruction.

The latest advancements between the years 2022 and 2024 have transformed the clinical utilization of low-field MRI through the employment of artificial intelligence-based techniques and modern hardware implementation. Recent research has demonstrated how Convolutional Neural Networks (CNNs), particularly U-Net and generative Adversarial Networks (GANs), have enhanced visual quality in low-field MRI. These advancements enable the effective implementation of DWI at lower field strengths, making low-field MRI a practical option in specific diagnostic areas, such as stroke and musculoskeletal imaging. Additionally, emerging evidence suggests its expanding applicability in other diagnostic domains, including lung imaging, fetal assessment,and neonatal brain imaging. This evolution of low-field MRI emphasizes the importance of appropriate patient selection and the development of novel regulations to maximize diagnostic yield.

## 2 TECHNOLOGICAL RESURGENCE

Conventionally, MRI systems operate at field strengths of 1.5 Tesla (T) and 3T, delivering exquisite spatial resolution but simultaneously requiring superconducting magnets and high-power infrastructure. Low-field MRI typically functions at field strengths below 1T, down to as low as 0.05T, which encourages essential advances in computational image reconstruction and hardware miniaturization to address its historical limitations in Signal-to-Noise ratio (SNR) and spatial resolution [3, 4]. The convergence of artificial intelligence-driven image enhancement and novel reconstruction algorithms has enabled current low-field systems to overcome the performance gap, making them viable alternatives to high-field systems.

## 3 DEMOCRATIZING ACCESS TO ADVANCED DIAGNOSTICS

The exorbitant cost of high-field MRI, ranging from $1 million to $3 million per unit, along with substantial operational overhead, has long been a disincentive to equitable access in low and middle-income countries [5]. Low-field MRI presents a transformative solution for decentralized and point-of-care imaging, with its significantly reduced capital expenditure, minimal shielding requirements, and lower power consumption. These compact devices facilitate installation in rural clinics, emergency settings, and mobile health units by eliminating reliance on liquid helium and high-power cooling systems, thereby narrowing the global diagnostic divide.

## 4 APPLICATIONS AS SHIFTING LANDSCAPE

Although traditionally dismissed due to alleged inadequacy in resolution, modern low-field MRI has demonstrated scientific efficacy across multiple domains, including neuroimaging, musculoskeletal diagnostics, and pulmonary imaging. In the sphere of stroke detection, recent studies indicate that ultra-low-field MRI (0.05T–0.1T) possesses promising sensitivity in recognizing ischemic strokes, offering a feasible substitute in emergency conditions [6, 7]. Additionally, for musculoskeletal evaluation, particularly in assessing soft tissue injuries and joint pathologies, low-field MRI provides adequate diagnostic precision at a lower cost, making it an attractive choice for sports and orthopaedic centers [8, 9]. A comparison between low-field and high-field MRI is shown in Table ****1****.

Paediatric applications also benefit from the reduced acoustic noise and portability of these systems. In NICUs, portable MRI has been successfully used for conditions, such as Intraventricular Hemorrhage (IVH) and Hypoxic-Ischemic Encephalopathy (HIE) [10]. In addition, emerging research supports its applicability in pulmonary imaging using short echo-time sequences, as well as in liver fibrosis staging and renal perfusion evaluation. Alongside stroke and musculoskeletal imaging, low-field MRI is also being explored for broader diagnostic use, including traumatic brain injury and tumor detection in low and middle-income countries (LMICs), where access to high-field MRI is limited.

## 5 BRIDGING THE GAP

The conjunction of scientific progression and cost-effectiveness positions low-field MRI as a significant facilitator in the healthcare sector. Companies such as Hyperfine, with their portable 0.064T MRI system, epitomize how disruptive innovation can overcome conventional limitations by offering imaging solutions that are not only economically feasible but also scalable across diverse healthcare settings. With the integration of deep learning into reconstruction methods, previous concerns regarding SNR have been further mitigated, reinforcing the idea that low-field MRI can serve as a valuable complement to traditional systems [11-13].

Emerging evidence suggests that in selected clinical contexts, such as acute stroke assessment and Musculoskeletal (MSK) examination, low-field MRI generally offers comparable diagnostic performance to high-field systems. Some studies have demonstrated sensitive and specific results in portable low-field stroke imaging compared to high-field MRI. Considering these findings, the results are encouraging, and low-field MRI should currently be regarded as a complementary modality rather than a replacement for high-field systems. Its role is particularly important where conventional MRI is inaccessible due to various constraints. To strengthen these claims, a comparative (Table ****2****) displaying head-to-head performance metrics, such as resolution, SNR, and scan time, has been presented, highlighting the key differences between low and high-field MRI systems.

## 6 FUTURE POLICY TRAILS

There is an urgent need for policy frameworks that promote the acceptance of low-field MRI in underserved regions as regulatory infrastructures evolve to accommodate this technological shift. Real-world efforts, such as the World Health Organization’s Global Atlas of Medical Devices, highlight the importance of equitable access to diagnostic tools. Additionally, initiatives by non-profit organizations in Africa are facilitating the deployment of portable imaging in underprivileged areas. Strategic collaborations among industry stakeholders, healthcare institutions, and government bodies will be critical to expanding deployment, training radiologists in interpreting low-field MRI images, and integrating these systems into existing clinical workflows. Furthermore, sustained investment in artificial intelligence-driven image processing, adaptive coil design, and innovative contrast methods will significantly enhance diagnostic accuracy, positioning low-field MRI not only as a complementary tool but also as an essential component of next-generation medical imaging [14]. Collectively, these innovations establish low-field MRI as a vital element of futuristic and decentralized medical imaging.

## 7 CHALLENGES AND ETHICAL CONCERNS

Despite its capabilities, low-field MRI is not without challenges. The comparatively lower spatial resolution, partially mitigated by artificial intelligence-driven improvements, still requires careful validation for critical applications, such as oncology, where high-fidelity imaging is paramount [15]. Furthermore, the smooth integration of low-field MRI systems into existing clinical frameworks demands thorough adaptation and rigorous training for radiologists unfamiliar with these modalities. While AI has enhanced image quality, noise suppression, and modalities like diffusion-weighted imaging (DWI) at lower field strengths, some limitations persist, including reduced Signal-to-Noise ratio (SNR), lower spatial resolution, and artifacts in patients with metallic implants. These factors can impact diagnostic yield and accuracy. Additionally, ethical concerns arise regarding the impartial distribution of imaging systems, especially in order to prevent economically deprived regions from completely relying on low-resolution imaging.

## 8 AI AND HARDWARE INNOVATIONS IN LOW-FIELD MRI

Emerging AI technologies have come into play to address limitations such as low Signal-to-Noise ratio (SNR), longer acquisition times, and reduced spatial resolution. Numerous AI-based architectures and networks have been developed to enhance image reconstruction. Methods, such as U-Net architecture, Generative Adversarial Networks (GANs), and Compressed Sensing (CS) are widely employed for segmentation, image restoration, artifact reduction, and accelerating MRI acquisition by reconstructing images from under-sampled data in low-field MRI. These advancements enable more accurate imaging of anatomical structures even at lower resolutions. Similarly, several hardware innovations, namely adaptive coil design, signal averaging, and parallel imaging, have improved signal detection efficiency and reduced acquisition noise in portable low-field MRI systems.

## 9 A NEW DAWN IN MEDICAL IMAGING

This shift towards low-field MRI represents a significant progression in medical imaging, emphasizing key aspects such as affordability, portability, and technical innovation. The global medical community stands at the forefront of a transformative era where diagnostic imaging is no longer restricted to high-cost, infrastructure-heavy facilities, thanks to the acceptance of this innovation. Low-field MRI has the potential to become the benchmark for primary healthcare units, emergency centers, and telemedicine initiatives, ensuring that state-of-the-art medical diagnostics transcend financial and geographical barriers.

## CONCLUSION

Low-field MRI has emerged as a promising, cost-effective alternative to conventional high-field systems. While this technology has few limitations related to image resolution, ongoing technological advancements continue to enhance its diagnostic capabilities. The future of low-field MRI demands sustained research, collaborative innovation, and strategic policy development. Nevertheless, the idea of impartial and universal imaging is now closer to becoming a reality than ever before.

## Figures and Tables

**Table 1:** Comparison of low-field and high-field MRI.

**Feature**	**Low-Field MRI**	**High-Field MRI**
**Image Resolution**	Moderate, enhanced with AI	High, with advanced imaging capabilities
**Portability**	Compact and can be placed on bedside	Stationary and heavy
**Deployment**	High in low and middle-income countries and rural regions	Limited due to infrastructure and cost
**Power Requirement**	Low and can operate on a standard power supply	High; typically requires a dedicated power supply
**Cost**	Low equipment and operating cost	High equipment and maintenance cost
**Scan Time**	Longer and improved with AI	Shorter time due to a stronger field

**Table 2:** Comparative overview of low-field *vs.* high-field MRI systems.

**Parameter**	**Low-Field MRI** **(0.064T – 0.5T)**	**High-Field MRI** **(1.5T – 3T)**	**Remarks**
**Signal-to-noise ratio (SNR)**	Lower (approx. lower than 1.5T)	High	Deep learning aids in SNR compensation
**Image Resolution**	Moderate (0.5–2 mm)	High	Limits the evaluation of subtle pathology
**Scan Time**	Longer (approx. 5–15 min)	Shorter (approx. 2–5 min)	Accelerated by AI-based reconstruction
**Diffusion-Weighted Imaging (DWI)**	Emerging capability	Standard	Stroke imaging is possible at low-field
**Cost**	Low (< $100,000 for some systems)	High (generally $1M–$3M)	Lower capital and maintenance cost
**Portability**	High (portable and bedside options available)	Fixed, non-portable	Enables bedside and emergency use
**Clinical Utility**	Good for large infarcts and bone, joint, and soft tissue injuries	Excellent for full spectrum and subtle ligament and cartilage detail	Portable at low-field; shown to be useful in stroke, MSK, pulmonary imaging, *etc.*

## LIST OF ABBREVIATIONS

MRI: Magnetic Resonance Imaging
DWI: Diffusion-Weighted Imaging
CNNs: Convolutional Neural Networks
AI: Artificial Intelligence
IVH: Intraventricular Hemorrhage
LMICs: Low And Middle-income Countries
HIE: Hypoxic-Ischemic Encephalopathy
GANs: Generative Adversarial Networks
CS: Compressed Sensing
SNR: Signal-to-noise Ratio
